# To what extent can clinical characteristics be used to distinguish encephalitis from encephalopathy of other causes? Results from a prospective observational study

**DOI:** 10.1186/s12879-018-3570-2

**Published:** 2019-01-22

**Authors:** Else Quist-Paulsen, Anne-Marte Bakken Kran, Elisabeth S. Lindland, Katrine Ellefsen, Leiv Sandvik, Oona Dunlop, Vidar Ormaasen

**Affiliations:** 1 Department of Infectious Diseases, Oslo University Hospital Ullevaal, P. O. Box 4956, Nydalen, N-0450 Oslo, Norway; 2 Institute of Clinical Medicine, University of Oslo, P.O Box 1171, Blindern, 0318 Oslo, Norway; 3 Department of Microbiology, Oslo University Hospital, P.O. Box 4956, Nydalen, N0450 Oslo, Norway; 4 Faculty of Medicine, University of Oslo, P.O Box 1078, Blindern, 0316 Oslo, Norway; 5 Department of Radiology and Nuclear Medicine, Oslo University Hospital, Rikshospitalet, Sognsvannsveien 20, N-0372 Oslo, Norway; 6 Sorlandet Hospital Arendal, Sykehusveien 1, N-4809 Arendal, Norway; 7 Department of Neurology, Oslo University Hospital, Ullevaal Hospital, P.O. Box 4956, N-0450 Oslo, Norway; 8 Oslo Group of Biostatistics and Epidemiology Oslo University Hospital, P.O. Box 4950, Sogn Arena, N-0424 Oslo, Norway; 9 Department of Acute Medicine, Oslo University Hospital, Ullevaal Hospital, P. O. Box 4956, Nydalen, N-0450 Oslo, Norway

**Keywords:** Infectious encephalitis, Encephalopathy, Diagnostic accuracy, Lumbar puncture, Central nervous system

## Abstract

**Background:**

Recognizing patients with encephalitis may be challenging. The cardinal symptom, encephalopathy, has a wide array of differential diagnoses. In this prospective study we aimed to explore the etiology of encephalitis and to assess the diagnostic accuracy of symptoms and clinical findings in patients with encephalitis in an encephalopathic population.

**Methods:**

Patients with acute onset of encephalopathy (*n* = 136) were prospectively enrolled from January 2014–December 2015 at Oslo University Hospital, Ullevaal. Clinical and biochemical characteristics of patients who met the case definition of encephalitis were compared to patients with encephalopathy of other causes.

**Results:**

Among 136 patients with encephalopathy, 19 (14%) met the case-definition of encephalitis. For 117 patients other causes of encephalopathy were found, infection outside the CNS was the most common differential diagnosis. Etiology of encephalitis was confirmed in 53% (4 bacterial, 4 viral, 1 parasitic, and 1 autoimmune). Personality change, nausea, fever, focal neurology, recent travel history, and low inflammation markers were significantly more abundant in patients with encephalitis, but the diagnostic accuracy for individual parameters were low (area under the curve (AUC) < 0.7). The combination of fever (OR = 6.6, 95% CI, 1.6–28), nausea (OR = 8.9, 95% CI, 1.7–46) and a normal level of ESR (erythrocyte sedimentation rate < 17 mm/hr, OR = 6.9, 95% CI, 1.5–33) was significant in multivariate analysis with an AUC (area under the curve) of 0.85 (95% CI, 0.76–0.94). Moderately increased pleocytosis in CSF (5-100 × 10^6^/L) further increased the diagnostic accuracy of this combination, AUC 0.90 (95% CI, 0.81–0.98).

**Conclusions:**

There is a wide diversity in differential diagnoses in patients with encephalopathy, and no single symptom or finding can be used to predict encephalitis with high accuracy in this group. The combination of fever, nausea and a low ESR in an encephalopathic population, increased the diagnostic accuracy of encephalitis compared to solitary parameters. The triad could be a useful clinical tool for early diagnosis of encephalitis, and these patients should be considered for further diagnostics such as lumbar puncture (LP).

**Electronic supplementary material:**

The online version of this article (10.1186/s12879-018-3570-2) contains supplementary material, which is available to authorized users.

## Background

Recognizing patients with infection in the central nervous system (CNS) is both challenging and important. Procedures to obtain prompt diagnosis and to initiate treatment of acute bacterial meningitis (ABM) are well established in most hospitals. In contrast, infectious or immune mediated encephalitis is a much less investigated condition. Alteration in mental function (encephalopathy) is the cardinal symptom of encephalitis. Encephalopathy has a wide range of causes, and encompasses several serious conditions in need of immediate evaluation and treatment, including encephalitis [1–3]. Herpes simplex virus 1 (HSV1) is considered the most common cause of sporadic encephalitis [3–7]. Early initiation of treatment with acyclovir has been shown to substantially reduce mortality and morbidity [8–12]. Other causes of encephalitis, such as *Mycobacterium tuberculosis* or immune-mediated encephalitis also benefit from early initiation of treatment [5, 13–17]. However, despite extensive testing, the etiology of encephalitis often remains unknown [4, 7, 13, 18–20]. In a retrospective study at our hospital, causative agent was confirmed in 42% of patients with encephalitis between 2000 and 2009 [21].

Identifying the encephalitic patient is the first vital step to initiate diagnostic algorithms and start empirical treatment. A lumbar puncture (LP) is mandatory, both to support the diagnosis and to ensure cerebrospinal fluid (CSF) for further analyses. Patients with encephalitis may present with subtle symptoms without typical symptoms of meningism. This may lead to delay in LP for patients suffering from encephalitis.

In this prospective study we wanted to explore the etiology and initial clinical presentation of patients with presumed infectious or autoimmune acute encephalitis. Secondly, we wanted to investigate whether clinical presentation or typical biochemical characteristics could be used to discriminate patients with encephalitis from patients with encephalopathy of other causes. Finally, we aimed to explore whether revised guidelines in the hospital have resulted in a higher identification rate of causative agents of encephalitis.

## Methods

Oslo University Hospital (OUS) is a local hospital for parts of the city of Oslo and regional hospital for 2,7 million people. Each year approximately 24,000 patients are admitted to the emergency room (ER). Adult patients admitted between January 2014 and December 2015 were eligible for this study, focusing on patients with encephalopathy. To be sure that no patient with encephalopathy was missed, we prospectively included all patients that (1) had onset of or worsening of central nervous system (CNS) symptoms within less than seven days, and (2) were examined by a LP. Thus, patients presenting with any symptom of meningism or mental change such as altered consciousness, changed personality, new onset epilepsy, new onset headache in combination with fever and focal neurological signs were eligible for inclusion.

**Fig. 1:**
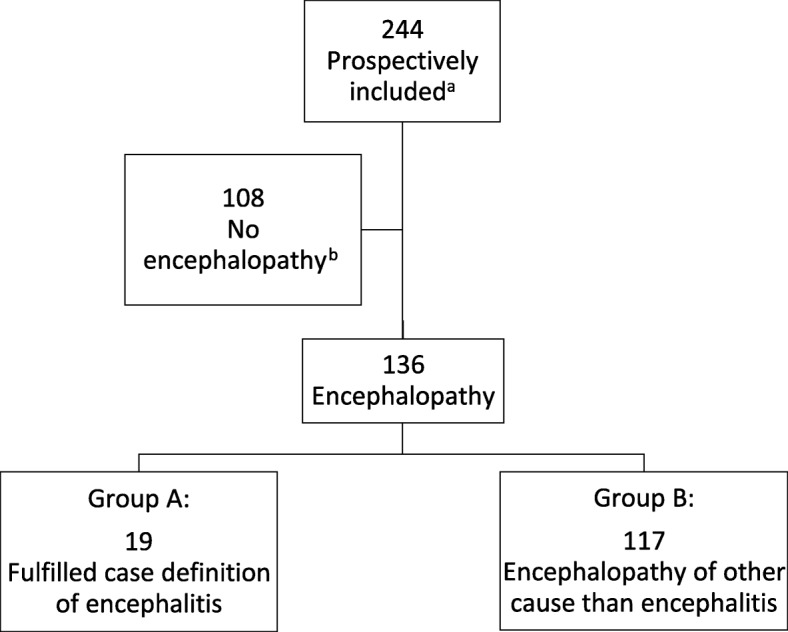
Inclusion of patients and study groups; Legend: a 272 patients met the inclusion criteria; for 28 patients no informed consent was obtained. b 108 patients in whom no encephalopathy was found were excluded from present study

Patients who did not fulfill the inclusion criteria of encephalopathy were excluded from further analyses (Fig. 1).

Encephalopathy was defined as a change in mental function according to the set of clinical criteria described in Table 1. The assessment of change in mental status was based on the story of the patient, family and/or treating physician.

**Table 1:** Case definitions

Encephalopathy	Decreased/altered consciousness (unconsciousness, the patient is hard to keep awake or extensively tiredness) *and/or* change in cognitive function (bewilderment, disorientation, latency, reduced ability to recall history or data) *and/or* personality change (aggression, new onset feeling of despair, inadequate/ strange behavior)
Encephalitis	Encephalopathy (=major criterion) for more than > 24 hrs, with no other cause identified and at least 2 of the following: Objective fever (≥38 °C) CSF WBC count ≥5 × 10^6^/L New onset seizures New onset of focal neurological findings CT/ MR findings consistent with encephalitis EEG findings consistent with encephalitis

Patients with encephalopathy were divided into two groups for comparisons: Group A (fulfilling the criteria of encephalitis), and group B (encephalopathy of other cause). The case definition of encephalitis (Table 1) was based on the same criteria and symptoms as stated in the International Encephalitis Consortium case definition [22] . Etiological agents for patients with encephalitis were classified using the criteria given in the review of Granerod et al. [14].

All patients were evaluated by neurologist or internist, and by the investigators within 0–2 days. Encephalopathy had to be present for at least 24 h to fulfill the main criteria of encephalitis. For all patients, prior medical history, travel history, symptoms and clinical findings on the day of the LP were recorded. Fever was defined as either ≥38 °C upon admission or within 24 h after admission, or measured to ≥38 °C by patient prior to admission. A feeling of fever without documentation of such, was not defined as fever due to the uncertainty/unreliability of this symptom.

Patients were regarded as immunocompromised if they were under treatment for or had been treated for cancer within the last year (including hematological malignancies), if they were diagnosed with HIV infection or diabetes mellitus type 2 (DM2) or if they were taking immunosuppressive or immune modulating drugs. Initial blood tests, findings from the LP, CT and/or MRI and/or EEG, and data on antimicrobial treatment as well as diagnosis at discharge were collected from the hospital records.

By including a patient in the study, the clinician could apply premade study packages consisting of a standard set of CSF analyses and serological screening for selected agents (see Additional file 1). All CSF samples were analyzed with in house real time polymerase chain reactions (PCR) for detection of herpes simplex virus 1 (HSV1) and 2 (HSV2), varicella zoster virus (VZV) and enterovirus. Microscopy and culturing for bacteria were performed according to routine procedures, and PCR analyses for common bacterial causes of meningitis were performed for patients treated with antibiotics prior to LP. Analyses for detection of other microbiological agents were performed if clinically relevant. Antibodies in serum and CSF were measured using serological assays in accordance with hospital recommendations. Biochemical analyses of blood and CSF were performed as part of the routine diagnostics using Sysmex XE-2100 (in 2014) and Sysmex XN-9000 (in 2015).

### Ethics and consent

All patients or next of kin were asked for informed consent before or shortly after inclusion. The study protocol was reviewed and approved by the The Regional Committees for Medical and Health Research Ethics (REC South East, reference number 2011/2578) and the ethical council of the hospital.

### Statistical methods

Patients fulfilling the case definition of encephalitis (group A) were compared to patients with encephalopathy of other causes (group B). Association between outcome variable and categorical data such as gender, immunosuppression, travel history and the presence of clinical symptoms were analyzed using Chi square test. Continuous data (e.g. age, blood pressure, heart rate, respiratory frequency and laboratory findings) were analyzed with Student t test for normally distributed data, otherwise Mann Whitney U test was used. Results are presented as mean (±SD) or median (interquartile range, IQR). Clinical symptoms and initial blood findings were combined in logistic regression analysis with encephalitis as dependent variable. Variables with more than 30% missing observations, e.g. D-dimer, were not analyzed as possible predictors for encephalitis. To measure how well a finding or model distinguishes between encephalopathy and encephalitis, the area under the ROC (receiver operating characteristic) curve (AUC) was calculated. The combination with the highest AUC was chosen as the final prediction model of encephalitis. In this final model, all parameters were significant when adjusting for each other.

Blood parameters were dichotomized to above or below reference levels of the hospital. For CSF findings, we found it neither relevant, nor safe to define a cutoff value for CSF parameters as the control group (group B) consists of cases with meningitis. Thus, for pleocytosis in CSF, intervals rather than absolute values were analyzed.

Statistical significance was defined as *p* < 0.05. Data were analyzed using IBM SPSS Statistics 24.

## Results

### Patient population

Two hundred seventy-two patients met the inclusion criteria for assessment of having encephalopathy. However 28 patients were not enrolled in the study as we were not able to obtain informed consent. For 108 patients no sign of encephalopathy was found and these were excluded from the study. 136 patients were considered to have symptoms of encephalopathy, of these 19 (14%) met the case definition of encephalitis (group A) as they fulfilled at least two minor criteria of the case definition, and no other cause of encephalopathy was identified. The diagnoses of patients in group B are shown in Table 2.

**Table 2:** Diagnoses in patients with encephalopathy of other cause than encephalitis (*n* = 117, group B)

Diagnostic category	No of patients
Infection outside CNS (*n* = 39)	
Respiratory tract	21
Urinary tract	3
Unknown/ other	15
Infection in CNS (*n* = 19)^a^	
Bacterial meningitis^b^	11
Aseptic meningitis^c^	5
Other CNS infections^d^	3
Neurological disease (*n* = 24)	
Epilepsy	13
Nonepileptic seizures	4
Other^e^	7
Encephalopathies (*n* = 8)^f^	
Toxic	4
Metabolic	4
Other diagnosis (*n* = 27)	
Cerebrovascular disorders^g^	6
Psychiatric disorders^h^	4
Unspecified disorientation and/ or coma	4
Malignancy in CNS^i^	3
Pulmonary embolism	3
Other^j^	7

^a^ Other than patients with encephalitis. ^b^ S.pneumonia (6), S.aureus (3), N meningitides (1), K.oxytoca (1). ^c^ Culture negative meningitis. ^d^ Neurosyfilis, possible H1N1 encephalopathy, intracerebral abcess. ^e^ Transient global amnesia (4), headache/migraine (3). ^f^ Alcohol withdrawal (2), drug intoxication (2), hepatic encephalopathy (2), hyponatriema (2). ^g^ Cerebral vasculitis (1), sinuous venous thrombosis (1), cerebrovascular accident (4). ^h^ Depression (1), psychosis (2), unspecified delirium (1). ^i^ Oligoastrocytoma (2), unknown (1). ^j^ Dementia with other conditions (2), inflammatory disease (2, Morbus Adult still and Hemophagocytic lymphohistocytosis), medication side effect (1), vestibularis neuritis (1), arrhythmia (1)

The most common condition was other infection, which was found in 58/117 (50%). Bacterial meningitis was confirmed in 11 patients by detection of bacterial agent by bacterial growth or PCR in CSF and/or blood culture findings.

The mean age (± SD) of the study population was 56.2 (±21) years. 47% were male. 15/136 patients were transferred to our center from other hospitals, 3 patients were transferred from the department of psychiatry.

### Etiology

Etiology was confirmed in 10/19 (53%) of patients with encephalitis. The cause of encephalitis in relation to number of minor criteria in the case definition is shown in Table 3. In 9 patients (50%), an infectious agent was confirmed (four bacterial, four viral, and one amoebic). Two patients had growth of *Mycobacterium tuberculosis* in the CSF, both presented without symptoms typical of meningitis. One of these had dual infection of TB and *Cryptococcus neoformans*. Specific IgG antibody response of *Borrelia burgdorferi* with elevated CSF/serum-index was found in two patients. None of these patients had headache or neck stiffness. The patient with *Naegleria fowleri* returned from holiday abroad on the day of admission and etiology was confirmed by sequencing of material from autopsy. One patient was diagnosed with autoimmune encephalitis with N-methyl-D-aspartate (NMDA) receptor antibodies in CSF. Another three study patients were suspected of having autoimmune encephalitis; two of these were treated with steroids and immunoglobulins.

**Table 3:** Etiology and number of minor criteria for patients diagnosed with encephalitis

Number of minor criteria	Etiology^a^	No of patients
2 minor criteria	Varicella zoster virus (VZV)^b^	2
	Adenovirus (ADV)^b^	1
	*Borrelia burgdorferi* ^c^	1
	*Naegleria fowleri* ^e^	1
	Unknown	2
≥3 minor criteria	Herpes simplex type 1^b^	1
	*Mycobacterium tuberculosis* ^d, f^	2
	*Borrelia burgdorferi* ^c^	1
	NMDAr	1
	Unknown	7

^a^Agent confirmed by either by ^b^ PCR in CSF, ^c^ intrathecal antibodyproduction or ^d^positive culture of *M tuberculosis* in CSF, ^e^ autopsy, sequencing of *Naegleria fowleri*. ^f^ One patient had dual infection with *Cryptococcus neoformans*

### Clinical characteristics of encephalitis (group a) compared to patients with encephalopathy of other cause (group B)

There were no statistical differences in age, gender, immunosuppression or duration of illness before admission, but patients with encephalitis reported more travel activity within the last 6 months (Table 4) compared with patients with encephalopathy of other cause.

**Table 4:** Clinical symptoms and findings of patients with encephalitis compared to patients with encephalopathy of other cause

Characteristics^a^	Encephalitis (group A)	Encephalopathy (group B)	*p*-value
Age, years	49 (±21)	57 (±21)	0.119
Gender, male	6/19 (32)	58/ 117 (50)	0.145
Duration of hospital stay, days	20 (10–37)	8 (4–17))	**0.001**
ICU	10/19 (53)	81/117 (69)	0.154
Death during hospitalization	3/19 (16)	10/117 (9)	0.326
No of patients with CNS symptoms < 24 hrs^b^	8/17 (47)	68/101 (67)	0.106
Immunodeficiency	4/19 (21)	28/ 117 (24)	0.784
Prodromal sickness^c^	14/19 (74)	75/109 (69)	0.670
Travel within 6 months	8/18 (44)	17/100 (17)	**0.009**
Headache	12/19 (63)	41/ 90 (46)	0.163
Photophobia	6/17 (35)	16/ 70 (23)	0.290
Nausea	13/16 (81)	35/ 81 (43)	**0.005**
Personality change^d^	9/ 19 (47)	25/105 (24)	**0.034**
Confusion^d^	15/19 (79)	62/106 (59)	0.091
Seizures^d^	5/18 (28)	29/ 105 (28)	0.989
Fever	14/19 (74)	55/117 (47)	**0.031**
Focal neurology	9/18 (50)	23/ 99 (23)	**0.019**
GCS^e^	14 (12–14)	13 (10–14)	0.219
GCS ≤14	16/18 (89)	90/114 (79)	0.324
GCS ≤8	1/18 (6)	27/114 (24)	0.080
Systolic blood pressure (mmHg)	140 (±28)	132 (±28)	0.275
Respiratory rate (/min)	19 (±6)	21(±7)	0.112
MRI findings suggesting encephalitis	8/17 (47)	1/63 (2)	**< 0.001**
EEG findings indicative of encephalitis	11/15 (73)	3/61 (3)	**< 0.001**

^a^ Data are presented as n/N (%), median (IQR) or mean (±SD). Significant differences in bold. ^b^ Patient’s description on how long they had been suffering from CNS symptoms before admission. ^c^ Prodromal sickness: symptoms before onset of CNS symptoms. ^d^ Observed/described either by patients, relatives or treating physician. ^e^ GCS at the day of LP, for 17 patients who were intubated before LP the GCS closest to intubation is registered, 4 patients had their LP > 24 h after intubation (=missing data). Also tested but not found to be relevant or not statistically significant: alcohol use, smoking habits, neck stiffness, illness among close relatives, vomiting, diastolic blood pressure, heart rate

Of the symptoms included in the major criterion of the case definition, a change in personality was significantly more abundant in patients with encephalitis compared to those with encephalopathy of other cause. The level of consciousness and Glasgow coma score (GCS) on the day of the LP was similar in both groups. Fever was present in 14/19 (74%) of patients with encephalitis, while only 55/117 (47%) of those with encephalopathy of other cause had fever. Focal neurologic deficit such as hemiparesis, aphasia and cranial nerve palsies were present in 9/18 of patients with encephalitis. Patients with encephalitis reported nausea more often than the comparison group.

### Biochemical findings

Biochemical laboratory results for group A and group B were compared, results are presented in Table 5. Median erythrocyte sedimentation rate (ESR), mean blood leucocyte count, mean neutrophil count and median D- dimer were significantly lower for patients with encephalitis.

**Table 5:** Comparison of laboratory parameters of encephalitis versus encephalopathy of other cause

Analysis	Encephalitis (group A)	Encephalopathy (group B)	*p*-value
Blood results^a^			
Leucocyte count (×10^9^/L)	9.1 (±4.0)	12.1 (±6.8)	**0.01**
- Leucocytes < 10 ×10^9^/L^b^	13/19 (68)	50/117 (43)	**0.037**
Neutrophil count (×10^9^/L)	6.8 (± 3.8)	10.4 (±6.6)	**0.002**
- Neutrophil < 7.3 ×10^9^/L^b^	12/19 (63)	34/98 (35)	**0.020**
ESR (mm/hr)	12.5 (3.0–26)	22.5 (9–46)	**0.015**
- ESR < 17 mm/hr^b^	13/18 (72)	41/100 (41)	**0.014**
CRP (mg/L)	6.3 (0.9–53)	32.0 (2.5–118.0)	0.09
D-dimer (μg/L, FEU)	0.6 (0.4–1.4)	1.9 (0.7–4.0)	**0.007**
CSF results^a^			
Leucocytes (×10^6^/L)	80 (22–153)	2 (1–5.5)	**< 0.001**
- CSF WBC 5–100 (×10^6^/L)	11/19 (58)	20/117 (17)	**< 0.001**
Protein (g/L)	0.87 (0.45–1.1)	0.39 (0.30–0.59)	**0.001**
- CSF protein > 0.45 (g/L)^c^	15/19 (79)	44/116 (38)	**0.001**
Glucose (mmol/L)	3.2 (±1.2)	4.1 (±1.8)	**0.023**
- CSF: blood glucose	0.5 (±0.2)	0.6 (±0.2)	0.071
Albumin (mg/L)	566 (295–747)	231 (147–345)	**0.005**
- CSF: blood albumin	13.6 (7.0–18)	6.3 (3.5–9.8)	**0.006**

^a^ data are median (IQR), mean (±SD) or n/N (%).^b^ No of patients with value below hospital reference level. ^c^ No of patients with value above hospital reference level. Analyzed, but not found relevant or significant; Hb, thrombocytes, monocytes, eosinophils, lymphocytes, sodium, potassium, creatinine, lactate dehydrogenase, ASAT, ALAT, INR, CK, total protein, albumin, and CSF opening pressure

**Table 6:** Comparisons between encephalitis (group A) and encephalopathy (group B)

Symptom/ finding	AUC	Univariate OR (95% CI)	*p*-value	Multivariate analysis (p, 95% CI)
Nausea	0.69	5.7 (1.5–22)	0.010	8.9 (p 0.010, 1.7–46)
Focal finding	0.63	3.3 (1.1–9.3)	0.024	
Fever	0.63	3.2 (1.1–9.3)	0.038	6.6 (p 0.011, 1.6–28)
Travel last 6 months	0.64	3.9 (1.3–11)	0.012	
ESR <17 mm/hr^a^	0.66	3.7 (1.2–11)	0.019	6.9 (p 0.014, 1.5–32)
Blood leucocytes <10×10^9^/L^a^	0.63	2.9 (1.0–8.2)	0.043	
Blood neutrophils <7.3×10^9^/L^a^	0.64	3.2 (1.1–9.0)	0.024	

^a^Hospital reference levels as described in methods

Median CSF WBC count and protein concentration was higher for patients with encephalitis. Differential count in CSF revealed polymorphonuclear (PMN) dominance in 2/19 (11%) patients with encephalitis, 12 patients from group B had PMN dominance > 70%, including all patients being categorized as bacterial meningitis.

### Predictors of encephalitis

Although some parameters differed significantly in patients with encephalitis compared with those with encephalopathy of other cause, no single symptom or finding showed good ability to predict the diagnosis of encephalitis (Table 6). ROC curves for selected single parameters are shown in Additional file 2.

**Fig. 2:**
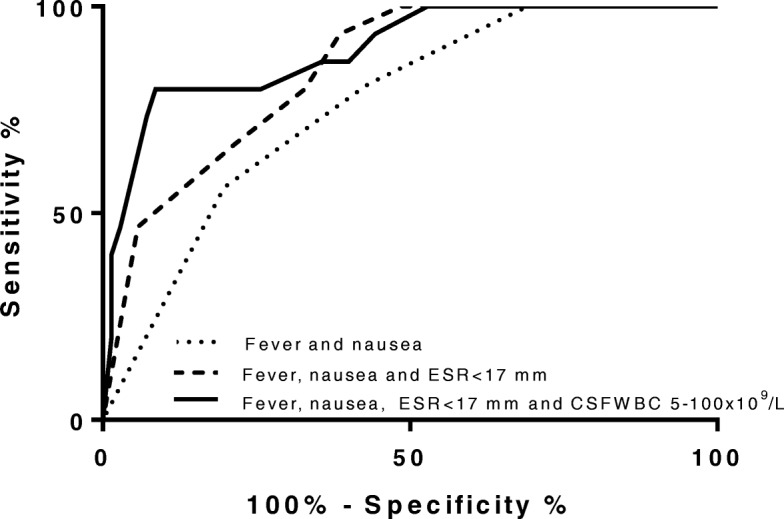
ROC curves for combination of clinical characteristics for the diagnosis of encephalitis in an encephalopathic population; Legend: Figure showing ROC curves of the model with the highest diagnostic accuracy of predicting encephalitis from regression analyses. AUC (95% CI) of predicted probabilities; fever and nausea: 0.76 (95% CI, 0.65–0.87); fever, nausea and ESR <17 mm/hr: 0.85 (95% CI, 0.76–0.94). When including the finding of CSF WBC of 5- 100 ×10^6^/L to the final model, an AUC of 0.9 (95% CI, 0.0.81–0.98) was reached

The best predictor among the blood parameters was ESR <17 mm/hr with AUC level of 0.66 (95% CI, 0.52–0.79). In CSF, WBC between 5 and 100 ×10^6^/L had an AUC of 0.70 (95% CI, 0.57–0.84). We wanted to assess whether combinations of symptoms or initial blood results could better predict the diagnosis of encephalitis. In regression analysis the combination of low ESR, fever and nausea had the highest AUC in this study (AUC 0.85 (95% CI, 0.76–0.94), Fig. 2). When choosing an optimal cutoff, this prediction model has a sensitivity and specificity of 80 and 67% respectively.

When entering a moderate increase in CSF WBC (5–100 × 10^6^/L) to the above mentioned model, the AUC increased to 0.90 (95% CI, 0.81–0.98) (Fig. 2). The respective OR for the variables in the latter model were 6.3 for fever (95% CI 1.3–31), 10.9 for nausea (95% CI, 1.7–70), 6.7 for ESR <17 mm/hr (95% CI, 1.3–35) and 8.9 for CSF WBC 5-100×10^6^/L (95% CI, 1.9–43).

## Discussion

Encephalitis is a serious condition with a high morbidity, and symptoms may be difficult to interpret. The diagnostic approach toward this disorder needs to be highly sensitive. In this observational study we demonstrate a wide range of differential diagnoses to encephalitis, including conditions warranting immediate treatment and follow up. Of all patients with encephalopathy only 19% were diagnosed with encephalitis, and of these 48% had no agent identified despite intensive diagnostic workup. We found that the combination of fever together with low ESR and nausea was the best predictor in discriminating encephalitis from other causes of encephalopathy: These symptoms combined were present in 47% (7/15) of patients with encephalitis, whereas only 6% (4/71) of the patients in the encephalopathy group had the same combination. Although the combination shows potential for predictive ability, it can neither exclude nor confirm encephalitis before LP. Nevertheless, the model makes use of easily accessible symptoms and blood findings that could be useful as a practical screening tool when considering lumbar puncture in an encephalopathic population.

Treatable causes of encephalitis were identified in more than half of the cases. The most notable finding was the high proportion of non-viral causes of encephalitis. Other studies report similar findings [4, 6, 15]. The study was carried out in a hospital with primarily local function, and only two of the patients were transferred from other hospitals. Surprisingly, HSV1, the main reason for empirical treatment of encephalitis with acyclovir, was detected in only one patient during the study period. Our findings indicate a broad spectrum of identified causes, and support the emphasis on targeted testing depending on previous exposure or travel history, as suggested by international guidelines [23, 24]. Furthermore, national and local surveillance of etiology of encephalitis should be stressed as etiology may change over time.

Considering the diversity in symptomatology, differential diagnoses and etiology of encephalitis, clinicians face a challenging task in identifying these patients. Optimal evaluation and treatment of patients with encephalitis depend on early recognition, and although patients with encephalitis may have no pleocytosis in their CSF [25–27], an LP should be performed without delay. Performing a LP is considered to be safe, as long as certain precautions are taken [28]. Our experience is that while the decision to perform a LP is straightforward if ABM is suspected, there is both delay and hesitation towards performing a LP for patients presenting with symptoms other than meningism. Lack of fever, no systemic sign of inflammation and other diagnosis being more probable, are arguments used against performing a LP. In this study we wanted to explore whether patients with encephalitis can be identified based on early clinical characteristics. Compared to patients presenting with encephalopathy of other causes, personality change, fever, focal findings, nausea and recent travel history were significantly more present in patients with encephalitis, but none of these findings alone did achieve a diagnostic accuracy of more than 70%. On the other hand, despite four patients with non-viral cause of encephalitis, the median levels of inflammation markers like ESR, leucocytes, neutrophils, CRP and D-dimer were significantly lower in patients with encephalitis. Thus, inflammatory blood parameters below reference level in patients with encephalopathy should by no means be used as an argument to abandon a LP. This finding is in accordance with a recently published study of Gennai et al., where a normal C-reactive protein in the absence of past neurological history together with normal/high blood pressure and seizure was proposed as a scoring tool for HSV encephalitis [29]. The association between fever and encephalitis in our study is not surprising as fever is integrated in the case definition, however five (25%) of the patients presented without fever. In four of these patients, etiology was confirmed (1 NMDAr, 2 with VZV, 1 with B. burgdorferi). Only one of these patients was immunosuppressed. Prediction models for encephalitis, similar to those proposed for bacterial meningitis, require larger studies [30–32]. Specific biomarkers for encephalitis would be very helpful in order to make a prompt diagnosis and initiate treatment. A search for biomarkers should be encouraged.

The identification of cause in 53% of our patients is an improvement compared to a retrospective study from the same hospital [21]. Increased focus on encephalitis, better diagnostic tools and/or more appropriate sampling on admission may be explanations for this. However, other studies have reported higher proportion of patients with known etiology (63–70%) [5, 6] .

The major strength of the present study is the clinically relevant and well-characterized control group based on our wide inclusion criteria in a daily life clinical setting, all considered to have some degree of encephalopathy. Recognition of the encephalitic patient, no matter cause, is mandatory for optimal evaluation as many patients with both infectious as well as immune mediated encephalitis benefit from early treatment. Therefore, we consider the heterogeneity in the causes of encephalitis observed in this study to be an advantage.

Clinical studies of rare conditions require large study populations, and the statistical power of the present study is relatively low due to the low number of cases with encephalitis, this implies that the validity of our findings may be limited. Furthermore, the confidence intervals are wide for some parameters. The case definition used in this study was based on a consortium definition which, due to clinical overlap, was formulated to capture both encephalopathy of presumed infectious etiology and encephalitis [22]. Patients having other conditions that could explain the state of encephalopathy, patients with encephalopathy <24 hours and patients in whom less than two minor criteria were fulfilled were strictly categorized “encephalopathy of other cause” (group B), including those with bacterial and aseptic meningitis [33]. Thus, some patients with encephalitis may have been misclassified.

## Conclusion

We found that the combination of nausea and fever together with a low level of ESR increased the probability of having encephalitis in an encephalopathic population. Normal inflammatory blood parameters and lack of fever cannot be used to defer or abandon LP. A wide diversity of etiological agents was found, and earlier studies showing herpes simplex as the most common cause of encephalitis was not confirmed in our material. We demonstrated a wide variety in differential diagnoses of encephalitis, many of which warrant immediate treatment. Larger clinical studies are needed to validate screening tools for encephalitis, and possible biomarkers need to be explored.

## Additional files

Additional file 1: Diagnostic evaluation of patients with suspected encephalitis (PDF 184 kb)

Additional file 2: ROC curves of relevant parameters showing AUC for predicting encephalitis in an encephalopathic population; Legend (in figure): AUC of probability of encephalitis for individual symptoms and findings, data from univariate logistic regression analyses. Figure 2a: personality change: 0.62 (95% CI, 0.47–0.76), focal findings: 0.63 (95% CI, 0.49–0.78), fever: 0.63 (95% CI, 0.50–0.76), travel: 0.64 (95% CI, 0.49–0.79), nausea: 0.69 (95% CI, 0.56–0.82). Figure 2b: ESR <17 mm/hr: 0.66 (95% CI, 0.52–0.79), blood leucocytes < 10 ×10^9^/L: 0.63 (95% CI, 0.50–0.76), blood neutrophils < 7.3 ×10^9^/L: 0.64 (95% CI, 0.51–0.78). Figure 2c: CSF WBC 5-100 ×10^6^/L: 0,70 (95% CI, 0,57-0,84), CSF protein > 0,45 g/L: 0,71 (95% CI, 0.59–0.83). (PDF 121 kb)

## Abbreviations

ABM: Acute bacterial meningitis
ADV: Adenovirus
AUC: Area under the curve
CNS: Central nervous system
CT: Computed tomography
DM: Diabetes mellitus
EEG: Electroencephalogram
ESR: Erythrocyte sedimentation rate
GCS: Glasgow coma scale
HSV1: Herpes simplex 1 virus
ICU: Intensive care unit
LP: Lumbar puncture
MRI: Magnetic resonance imaging
NMDA: N-methyl-D-aspartate
PCR: Polymerase chain reaction
ROC: Receiver operating characteristics
TB: Mycobacterium tuberculosis
VZV: Varicella zoster virus
WBC: White blood cell count
