# Supplementary material for: To what extent can clinical characteristics be used to distinguish encephalitis from encephalopathy of other causes? Results from a prospective observational study
Source: BMC Infect Dis. 2019 Jan 22;19:80. doi: 10.1186/s12879-018-3570-2 (PMC6343342; doi:10.1186/s12879-018-3570-2)
Supplement: Supplementary file 1 — Diagnostic evaluation of patients with suspected encephalitis (PDF 184 kb) [file 12879_2018_3570_MOESM1_ESM.pdf]

Additional file 1: Diagnostic evaluation of patients with suspected encephalitis

Step 1 Initial testing, all patients[a]

CSF ("study package"):

- Leucocyte count, protein, glucose, albumin
- Virus PCR: enterovirus, HSV1 and 2, VZV
- Microscopy and culture; if treated with antibiotics; bacterial PCR
- Extra CSF stored for additional testing [a,b]

Serum ("study package"):

- HIV, EBV, CMV, Parvo B19 virus, Toxoplasmosis, Treponema
- Extra serum stored for additional testing [a,b]

Other tests (recommended):

- noseswab (airway viruses, mycoplasma pneumoniae)
- faeces for enterovirus
- vesicles (HSV-/VZV-PCR)

If negative or clinical suspicion of auto- immune encephalitis:

- Encephalitis antibodies in serum and CSF[b]
- Paraneoplastic antibodies in serum and CSF[b]
- Screening for malignancy (CT scan, tumor markers)

Step 2 If step 1 is negative: repuncture

- repeat PCR analysis of HSV, EV, VZV
- antibody production of HSV, VZV in CSF and serum
- consult infectious disease specialist for further analysis

[a] **Special indications:**

- if travel abroad: discussion with infectious disease consultant and targeted testing depending on destination and incubation period
- if immunocompromised: JC virus, Cryptococcus (antigendetection and PCR), HHV6, listeria monocytogenes, CMV- PCR, EBV-PCR
- Possible tick exposure (seasonal): B burgdorferi antibodies in serum and CSF and Tick borne encephalitis in serum.
- Exposure to TB or travel/ stay in TB endemic areas; CSF smear for acid fast bacilli, CSF mycobacterial culture, MTB PCR for CSF, Quantiferon

[b] **CSF and serum;**

Encephalitis antibodies: NMDAr, AMPAR1 and 2, GABARB1, CASPR2, LGI1, +/- VGKC

Paraneoplastic antibodies: Hu, Ri, Yo, amphiphysin, CRPM5, MA1, Ma2
