# Supplementary material for: To what extent can clinical characteristics be used to distinguish encephalitis from encephalopathy of other causes? Results from a prospective observational study
Source: BMC Infect Dis. 2019 Jan 22;19:80. doi: 10.1186/s12879-018-3570-2 (PMC6343342; doi:10.1186/s12879-018-3570-2)
Supplement: Supplementary file 2 — ROC curves of relevant parameters showing AUC for predicting encephalitis in an encephalopathic population; Legend (in figure): AUC of probability of encephalitis for individual symptoms and findings, data from univariate logistic regression analyses. Figure 2a: personality change: 0.62 (95% CI, 0.47–0.76), focal findings: 0.63 (95% CI, 0.49–0.78), fever: 0.63 (95% CI, 0.50–0.76), travel: 0.64 (95% CI, 0.49–0.79), nausea: 0.69 (95% CI, 0.56–0.82). Figure 2b: ESR <17 mm/hr: 0.66 (95% CI, 0.52–0.79), blood leucocytes < 10 ×109/L: 0.63 (95% CI, 0.50–0.76), blood neutrophils < 7.3 ×10^9^/L: 0.64 (95% CI, 0.51–0.78). Figure 2c: CSF WBC 5-100 ×106/L: 0,70 (95% CI, 0,57-0,84), CSF protein > 0,45 g/L: 0,71 (95% CI, 0.59–0.83). (PDF 121 kb) [file 12879_2018_3570_MOESM2_ESM.pdf]

Additional file 2:

ROC curves of relevant parameters showing AUC for predicting encephalitis in an encephalopathic population

2A: ROC curves of individual symptoms/ findings

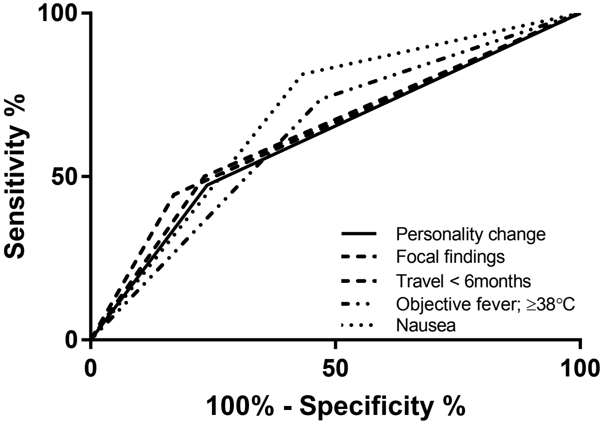

2B: ROC curves of individual blood analyses

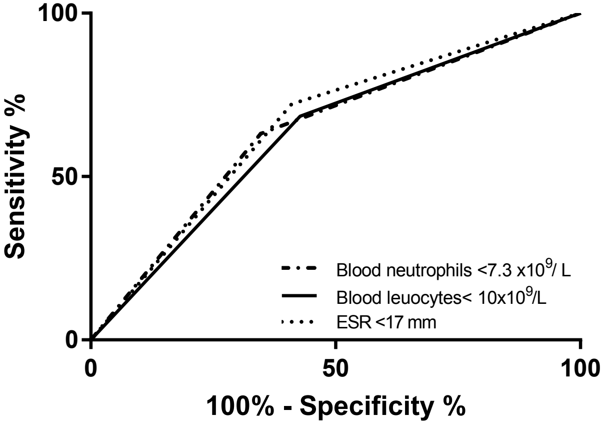

2C: ROC curves of CSF findings

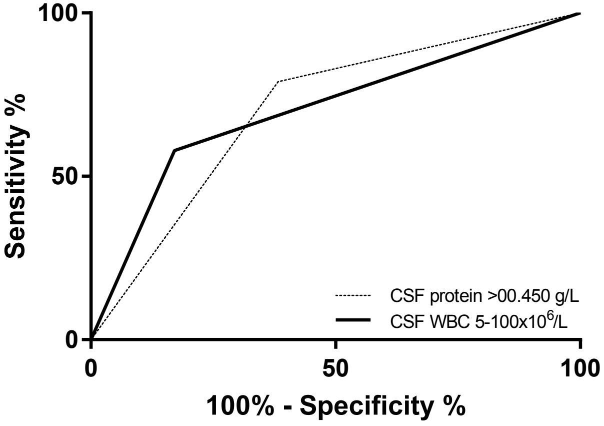

AUC (95% CI) of individual symptoms and findings, data from univariate logistic regression analyses.
Fig 2A: personality change: AUC= 0.62 (95% CI, 0.47-0.76), focal findings: AUC = 0.63 (95% CI, 0.49-0.78), fever: AUC = 0.63 (95% CI , 0.50-0.76), travel : AUC = 0.64 (95% CI, 0.49-0.79), nausea: AUC = 0.69 (95% CI , 0.56-0.82). **Fig 2B**: ESR<17 mm: AUC = 0.66 (95% CI , 0.52-0.79), blood leucocytes <10x10 ^{9}/L: AUC = 0.63 (95% CI, 0.50-0.76), blood neutrophils <7.3x10 ^{9}/L: AUC = 0.64 (95% CI, 0.51-0.78). **Fig 2C**: CSF WBC 5-100x10^6/L: AUC = 0,70 (95% CI, 0,57-0,84), CSF protein >0,45 g/L: AUC = 0,71 (95% CI, 0.59-0.83).
